# Chromatic aberration and spectral dependency of extended-range-of-vision intraocular lens technology

**DOI:** 10.1038/s41598-023-41634-z

**Published:** 2023-09-07

**Authors:** Grzegorz Łabuz, Weijia Yan, Isabella D. Baur, Ramin Khoramnia, Gerd U. Auffarth

**Affiliations:** The David J. Apple Center for Vision Research, Department of Ophthalmology, University Hospital Heidelberg, Im Neuenheimer Feld 400, 69120 Heidelberg, Germany

**Keywords:** Optical techniques, Translational research

## Abstract

This study compared the optical quality and chromatic performance of refractive-diffractive intraocular lenses (IOLs) that are designed to extend the range of vision of pseudophakic patients and alter chromatic aberration. Five IOLs were evaluated, Tecnis Synergy and Triumf POD L GF, both intended to compensate for eye's chromatism, as well as Acriva Trinova Pro C—a lens that increases chromatic aberration, and AT Lisa Tri and AcrySof IQ PanOptix. An optical setup composed of a corneal model inducing monochromatic and chromatic aberrations and incorporating various spectral conditions was employed. The two chromatic-aberration correcting IOLs demonstrated the lowest far-focus dispersion, but it was negative only, with the Synergy indicating its ability to reduce eye’s chromatic aberration. Although the Trinova increased far-point chromatism, it was close to the level of the PanOptix, but higher than that of the AT Lisa. All the studied models demonstrated varying optical quality in response to light color. Still, the strongest spectral dependency was associated with achromatizing technology. Therefore, chromatic aberration and wavelength dependency should be considered in IOL optimization and predicting visual function, particularly in non-white spectral conditions.

## Introduction

The contribution of the ocular media to longitudinal chromatic aberration (LCA) and its impact on visual function has been extensively studied over the past decades^1–3^. Although most of the available data come from phakic eyes, it has been later noted that crystalline lens extraction and implantation of an intraocular lens affect the eye's dispersion^4^. Differences between various IOL models have been identified, which resulted from their intrinsic material properties^4–7^. Later, the concept of engineering the eye's chromatic aberration through a hybrid refractive-diffractive IOL was proposed, which was first implemented in a low-power extended-depth-of-focus element^8^. Recently, the management of the eye's dispersion has also been implemented in trifocal technology^9–11^, but available literature on their achromatic function seems incomplete.

Tecnis Synergy and Triumf POD L GF are recently introduced presbyopia-correcting IOLs^9–11^, which also feature technology reducing the impact of the eye's LCA. Several clinical reports on the visual function of these two models can be identified^9–11^; none, however, addresses the issue of chromatic correction or spectral dependency. Still, it can be understood, given the challenges associated with in vivo testing of chromatic performance and the extensive nature of such examinations^12^. In this context, applying an optical bench to study the impact of the IOL design on the eye's chromatism appears suitable^8,12–14^. Due to recent advancements in establishing the link between optical quality and visual function, this approach can also be used to simulate postoperative visual acuity (VA)^15,16^. The adaptation of measurement conditions mimicking the eye and the development of non-linear formulas to predict the clinical effect are the main contributors that improved the agreement between laboratory and in vivo results, as discussed in a recent study^17^. Therefore, this approach can expand our knowledge of the function of this latest trend in IOL technology.

LCA-correcting IOLs often use two diffractive orders, typically 1st and the 2nd, to reduce dispersion effects at the free foci^8,13,18^. However, other designs have been introduced, which may yield an increase of chromatic aberration as a result of incorporating negative orders, such as Trinova's -1st diffractive order used for distance vision, but the implications of this approach have yet to be assessed. Although more data can be found on established trifocals, including their spectral dependency^14^, the potential clinical impact of changes in their performance when different than a design wavelength is used requires further research.

In this study, the LCA and the polychromatic performance of achromatizing IOLs and one that effectively increases chromatic aberration were evaluated. The comparison with established trifocals by means of optical-quality metrics and simulated visual function was also performed.

## Methods

### Intraocular lenses

Chromatic effects were studied in the following diffractive-refractive models, Acriva Trinova Pro C Pupil Adaptive (VSY Biotechnology, Turkey), Acrysof IQ PanOptix (Alcon, USA), Tecnis Synergy IOLs (Johnson & Johnson Vision, USA), AT LISA Tri 839MP (Carl Zeiss, Germany) and FineVision Triumf POD L GF (PhysIOL, Belgium).

Table 1 presents the optical characteristics of the studied lenses. The Trinova and the AT Lisa are made of hydrophilic material showing the lowest dispersion. Although the Synergy is made of a hydrophobic polymer, its refractive index and the Abbe number are close to that of the hydrophilic counterparts. By contrast, Triumf's hydrophobic material induces a higher LCA, followed by PanOptix's AcrySof, with the lowest Abbe number among the studied lenses.

**Table 1:** Design and material characteristics of the trifocal IOLs. Ref. Ind. = refractive index; Abb. No. = Abbe number; Int. = intermediate; NA = not available; Z[4,0] = primary spherical aberration.

	Trinova Pro C	PanOptix	Synergy	AT Lisa	Triumf
Material					
Ref. Ind.	1.46	1.55	1.47	1.46	1.53
Abb. No.	58	37	55	58	42
Add power					
Int. [D]	1.8	2.17	–	1.66	1.75
Near [D]	3.6	3.25	–	3.33	3.50
Diffractive orders					
Far	− 1	0	NA	0	+ 1
Int.	0	2	NA	+ 1 (1st profile)	+ 2 (1st profile)
Near	+ 1	3	NA	+ 1 (2nd profile)	+ 2 (2nd profile)
Spherical-aberration correction					
Z[4,0]	− 0.10 µm	− 0.10 µm	− 0.27 µm	− 0.18 µm	− 0.11 µm

In addition to material dispersion, the design of a diffractive pattern also contributes to the total chromatic dispersion of the eye^8,13,18^. This approach has been used to reduce the LCA of eyes implanted with extended-depth-of-focus lenses and is also applied in the Synergy and Triumf technology.

The IOLs differ in their light energy distribution intrinsic to their optical design. While the loss of light caused by diffractive optics has been well described^19–21^, IOL manufacturers often provide information about the light partition percentage related to the light that contributes to the desired image formation. Despite substantial advancements in minimizing light loss^20^, it is essential to recognize that none of the available technologies achieve 100% efficiency in light energy utilization. The Acriva employs 'Pupil Adaptive' technology, which modulates the light split between three foci. As a result, at 3 mm, the lens distributes 43% of available light to far, 21% to intermediate, and 36% to near foci. But the lens behavior changes as the aperture increases with the dominance of the intermediate focus over the near one. The Triumf also reinforces the intermediate range with 30% of allocated energy compared to 20% at near. A reversed proportion can be observed in the AT Lisa, but for the PanOptix, it is 25% for each secondary focus. Neither Synergy's energy distribution nor power additions have been specified by its manufacturer.

Given the extensive nature of the assessment and the high repeatability and reproducibility of modern IOLs^17,18,22^, only one sample was tested for the optical-quality comparison. All IOLs had a nominal power of + 20D.

### Optical testing

**Figure 1:**
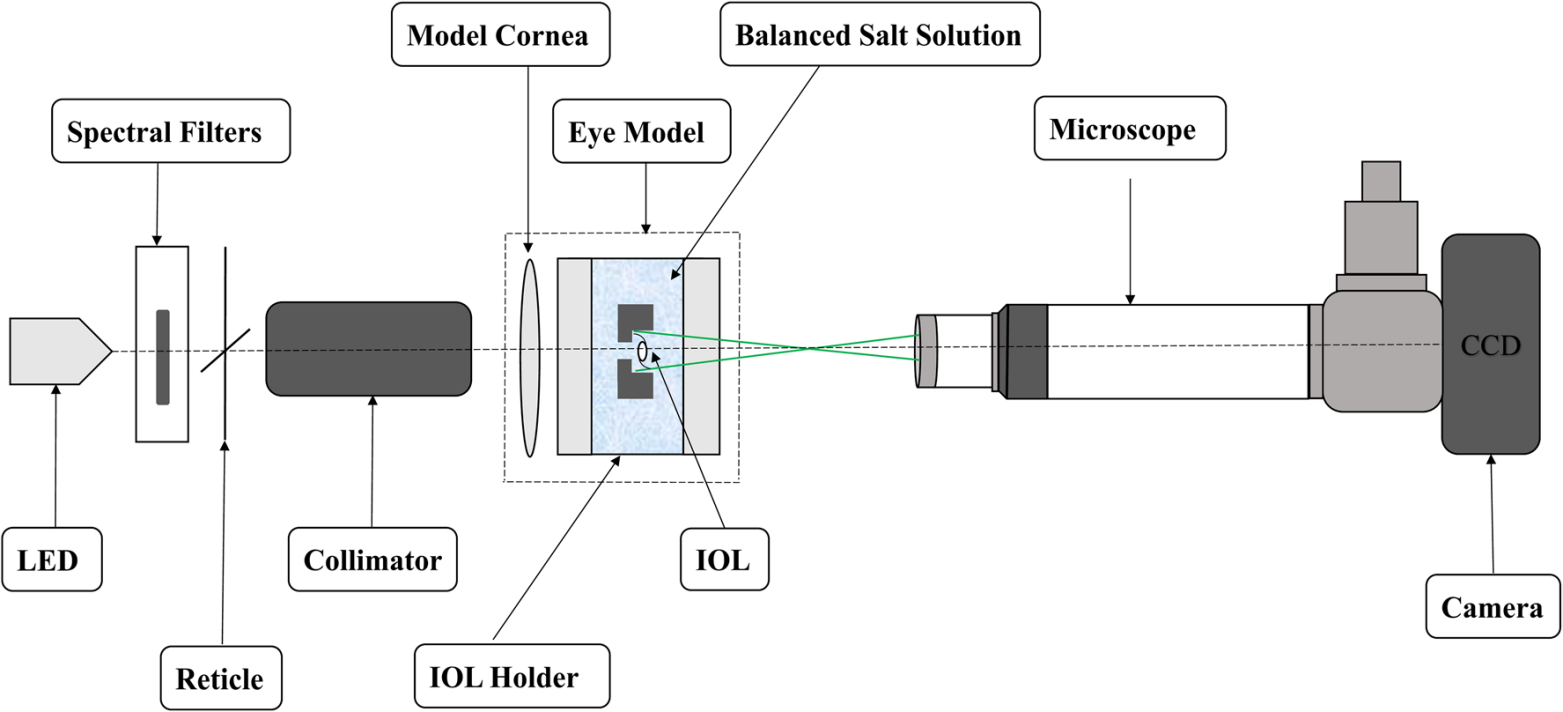
Schematic drawing of the optical-metrology setup. LED = light-emitting diode; CCD = charge-coupled device; IOL = intraocular lens.

The trifocal models were assessed with OptiSpheric IOL PRO2 (Trioptics GmbH, Germany) developed for testing IOL compliance with ISO-11979. A schematic diagram of the device is presented in Fig. 1. The measurements were performed using a singlet yielding + 0.27 µm of spherical aberration (SA) at 5.15 mm to mimic the human cornea. A polychromatic light source and a set of filters to test the IOLs in blue (480 ± 10 nm), green (546 ± 10 nm), and red (644 ± 10 nm) light. In addition, a filter that simulates the spectral sensitivity of a photopic eye was applied; hence, four spectral conditions were assessed in the current study.

The LCA of the IOLs was obtained from the difference between the IOL's power measured without a corneal model in red (644 ± 10 nm), green (546 ± 10 nm), and blue (480 ± 10 nm) light, and it was expressed in diopters according to the formula:

$$LCA = IOL power_{480} - IOL power_{644} \left[ D \right]$$

The LCA curves were centered at 546 nm, and then the second-order polynomial fit of LCA data was applied. The LCA of the corneal lens (i.e., aphakic condition) applied in this study was 1D^13^.

The modulation transfer function (MTF) was obtained in polychromatic and monochromatic light (480 nm, 546 nm, and 644 nm). A 50 lp/mm MTF criterion was applied to find the best focus as stipulated by ISO-11979. Then, sagittal and tangential MTFs were acquired at each focal point in three measurements, and the two meridians were averaged. The area under the MTF (MTFa) was calculated and used for VA simulations as described elsewhere^16^. Note that the multi-color simulations assumed the sole MTF effect without considering the eye spectral sensitivity and were used to facilitate comparative analysis. The optical assessment was performed at 3.5 mm due to difficulties in detecting the intermediate point of PanOptix at lower apertures^17^. Since at higher apertures, chromatic effects are confounded by spherical aberration, posing a challenge in identifying individual foci and increasing the measurement uncertainty^23^, only one pupil size was applied in this study.

## Results

### Longitudinal chromatic aberration

**Figure 2:**
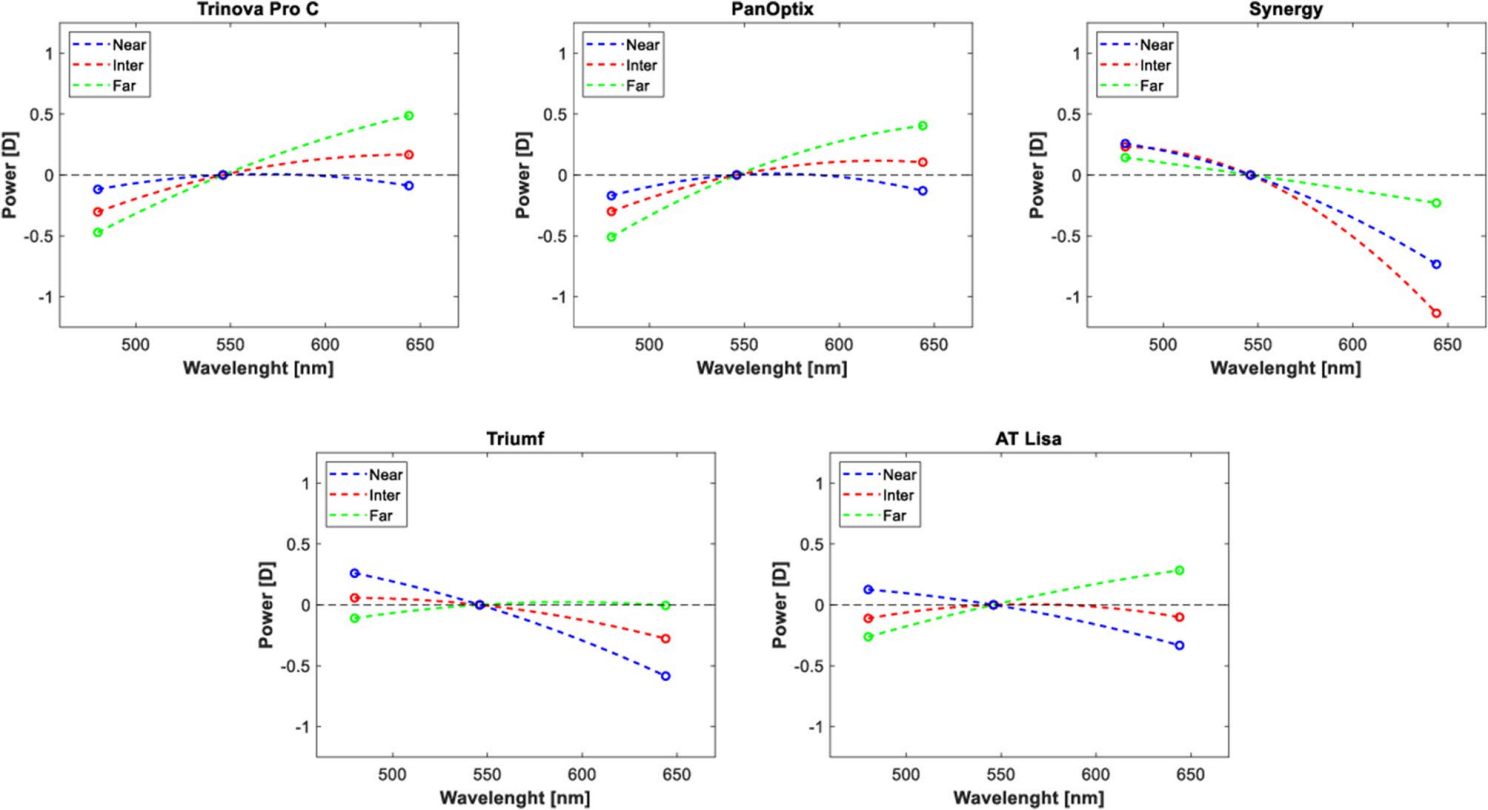
The longitudinal chromatic aberration (LCA) of the trifocal models at the three designed foci (IOL plane). The dashed line indicates the polynomial fit of degree 2.

Figure 2 shows the LCA for five lenses and the three foci. The chromatic focus shift was measured at 480 nm and 644 nm and is presented in Table 2. The Trinova demonstrated the largest LCA at the far-point. By contrast, Triumf's LCA was nearly corrected at far and overcorrected with the Synergy. At intermediate, Synergy's overcorrection substantially increased and was followed by a slightly negative LCA of the Triumf. The AT Lisa's LCA was virtually zero, but that of the remaining implants was above this level and ranged from 0.41D to 0.47D. The Trinova's and PanOptix's near LCA were nearly corrected—the two lenses showed close LCA results at all three foci as opposed to the Synergy, the AT Lisa, and the Triumf, which exhibited negative LCA values.

**Table 2:** Longitudinal chromatic aberration (LCA) of the studied IOLs at the three designed foci. The LCA is the difference between the nominal power measured at 480 nm and 644 nm. All reported values correspond to the IOL plane. Int. = intermediate.

Model	Trinova Pro C	PanOptix	Synergy	AT Lisa	Triumf
Far [D]	0.96 ± 0.00	0.92 ± 0.02	− 0.37 ± 0.07	0.55 ± 0.01	0.10 ± 0.01
Int. [D]	0.47 ± 0.00	0.41 ± 0.02	− 1.37 ± 0.04	0.01 ± 0.03	− 0.34 ± 0.05
Near [D]	0.03 ± 0.07	0.04 ± 0.00	− 0.99 ± 0.03	− 0.46 ± 0.00	− 0.85 ± 0.01

#### Optical-quality assessment

**Figure 3:**
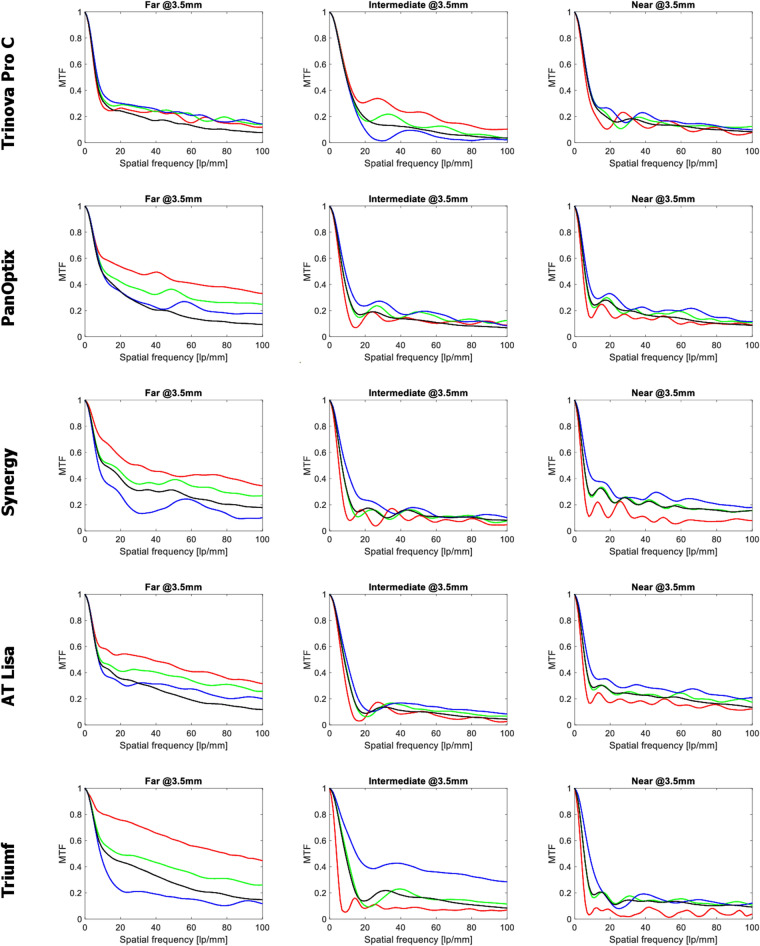
MTF levels of the studied IOLs at the best far, intermediate, and near focus compared for each spectral condition—480 nm (blue), 546 nm (green), 644 nm (red) and polychromatic light (black).

The MTF curves derived at the best focus for monochromatic and polychromatic conditions are compared in Fig. 3. Note that the optical quality was assessed with the cornea model, which increases the net LCA level as compared to Fig. 2 and Table 2. At far, PanOptix's MTF value loss at 50 lp/mm between green and polychromatic conditions was 49%. By contrast, the Synergy presented the lowest decrease of the MTF (21%), which was followed by the Triumf (29%), the Trinova (34%), and the AT Lisa (38%). All IOLs demonstrated a lower MTF loss in polychromatic conditions at the intermediate point, which ranged from 5% (Triumf) to 33% (PanOptix). At the near focus, the PanOptix showed a 13% MTF reduction. A close result was obtained with the Trinova, which showed 11%. Virtually no change in the optical quality of the AT Lisa, the Triumf, and the Synergy was observed in polychromatic conditions.

Except for the Trinova, all the studied models exhibited a high spectral dependency with an overall improvement of the MTF in red compared to blue light at far focus. The observed MTF increase at 50 lp/mm was 1.6-fold in the AT Lisa, 1.8-fold in the PanOptix, twofold in the Synergy, and 3.6-fold in the Triumf. By contrast, the MTF of the Trinova was minimally better in blue than in red light. However, it was reversed at the intermediate point with a 2.7-fold higher 644-nm MTF at 50 lp/mm. The blue-light condition MTF was between 1.5- and 1.6-fold higher than in the red-light condition in the AT Lisa, the PanOptix, and the Synergy. The most substantial difference (i.e., 4.4-fold) was observed in the Triumf with a better 480-nm performance, which was close to 4.2-fold recorded at the near-point with the same model. A close correspondence between red- and blue-light MTFs was observed in the Trinova at the best near focus. The PanOptix and the AT Lisa revealed 1.3–1.4-fold better 480-nm performance; for the Synergy, it was 2.7-fold.

**Figure 4:**
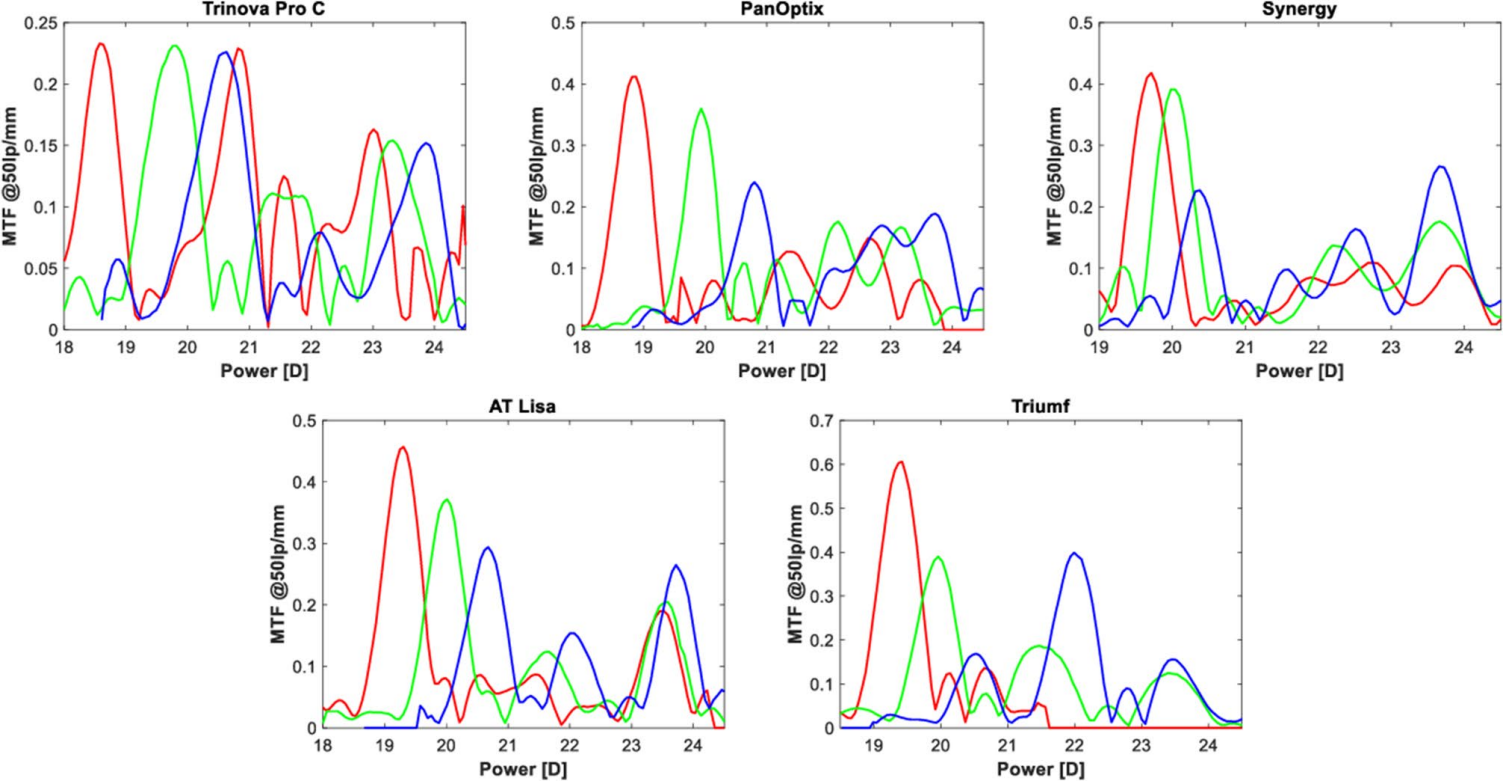
Through-focus (TF) MTFs of the trifocal IOLs measured at 480 nm (blue), 546 nm (green), and 644 nm (red). The polychromatic TF MTF was omitted to enhance graph readability. Note the difference in scale between the subfigures.

The through-focus MTF at 50 lp/mm measured at the IOL plane with the corneal model is presented in Fig. 4. The foci separation results from the combined effect of IOL's LCA and the model-eye contribution. The Trinova demonstrated three major peaks in all spectral conditions, with an additional peak in red light reinforcing the intermediate range. Similar to Fig. 2, only a slight difference can be noted in the height of the primary peak between the three colors of the Trinova. The Synergy's near MTFs overlap, indicating a progressive separation of foci at intermediate and far points. The spectral dependency of the optical quality is particularly noticeable in this model, as well as in the remaining lenses. The Triumf appears most affected, with a close-to-zero performance at 50 lp/mm in red light at a reading distance.

**Figure 5:**
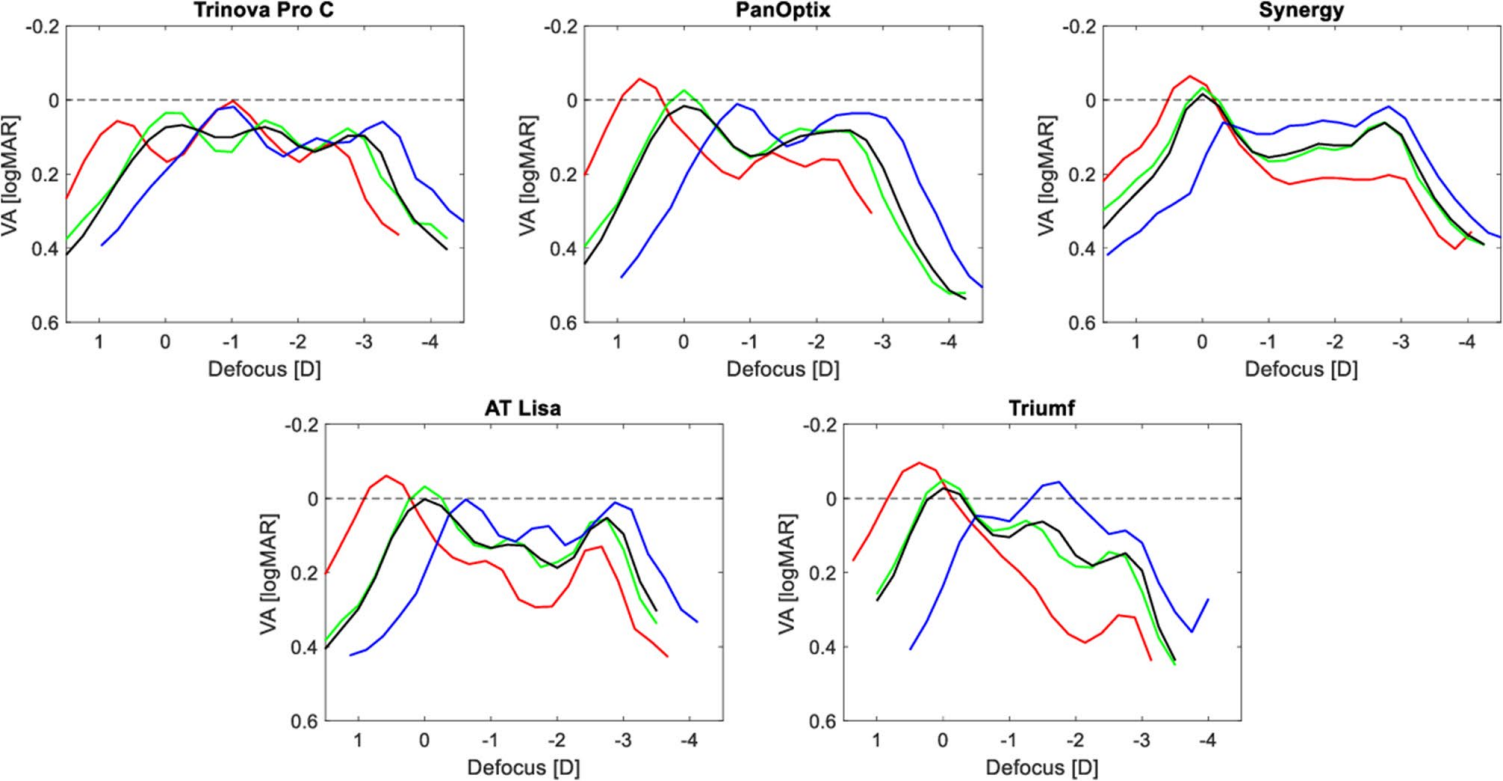
Simulating visual acuity changes with defocus (spectacle plane) measured in 480 nm (blue), 546 nm (green), 644 nm (red), and polychromatic (black) light. The dashed line indicates 0.00 logMAR.

The defocus-curve simulations of the trifocal IOLs under the studied spectral conditions are presented in Fig. 5. The Trinova showed a minimal VA change of ~ 0.02 logMAR (one optotype) at the peak of each focus. However, at no defocus (corresponding to the primary peak of the 546 nm line), a VA reduction may be more substantial—about one logMAR line in the Trinova. However, its LCA improved the intermediate range given the position of the red- and blue-light foci between the designed far and intermediate focus of the green line. The other models revealed a classic pattern of decreasing far VA from 644 nm through 546 nm to 480 nm, with the highest predicted value in red light. On the other hand, the intermediate and near focus was consistently improved in blue light and reduced in red light, which was particularly pronounced in the Triumf and the Synergy models. Both demonstrated a nearly two-line (logMAR) difference in predicted VA at the reading distance between blue- and red-light conditions, with the most substantial difference (i.e., ~ 0.4 logMAR) observed at the intermediate range of the Triumf. Still, the Synergy demonstrated a closer overlap of the green and black lines representing the 546-nm and polychromatic conditions, respectively.

## Discussion

We demonstrated that the monochromatic performance of trifocal IOLs may differ from the polychromatic when utilizing wavelengths from both extremes of the visible spectrum. Additionally, LCA effects may exacerbate the spectral dependency by introducing defocus blur affecting polychromatic image quality. A broad range of LCA (from negative to positive) was found, which is material- and design-related.

The LCA of the phakic eye is caused by light dispersion in ocular media, such as the cornea and the crystalline lens^1–3^. After replacing the natural lens with a monofocal IOL, the eye's chromatism is modified according to the dispersion properties of the IOL material (quantified by its Abbe number)^4–7^. Reports of the Abbe number of different IOL biomaterials range from 35 to 60^7^. In principle, the higher the value, the lower the LCA. Table 1 summarizes the Abbe numbers of the trifocal IOLs that influence their refractive-base LCA. The highest-dispersion properties of the analyzed materials had AcrySof IQ from Alcon. An SN60WF lens made of the same material demonstrated an LCA of 0.82D in our earlier analysis, indicating close correspondence to the far-focus LCA of the PanOptix^13^. In that study, a CT Asphina IOL (Carl Zeiss, Germany) was also included and showed the 0.37D chromatic-power difference. Since the CT Asphina served as a refractive base for its trifocal counterpart—AT Lisa Tri, only a slight difference was observed at far. Although we could not identify a prior study reporting the LCA of Acriva monofocal IOLs, the Trinova has the identical Abbe number and refractive index as the AT Lisa, allowing for the assumption of similarities between the two refractive platforms. This was confirmed by the close relation between the far-focus LCA of the AT Lisa and the intermediate LCA of the Trinova—both reflecting the contribution of the refractive base alone. The current setup was also applied to measure the dispersion of a Tecnis material (Łabuz et al., 2023 ASCRS annual meeting in Washington, DC, USA), of which the Synergy refractive part is composed. We found an LCA of 0.42D, which indicates Synergy’s ability to lower the eye’s dispersion, as we recorded negative values at all foci. It was, however, not the case for the Triumf, which showed low albeit positive LCA at far focus. This can be explained by a higher dispersion of its hydrophobic material, given its monofocal predecessor (PODEYE, PhysIOL, Belgium) yielded an LCA of 0.71D.

In addition to material dispersion, the applied diffractive orders also affect the chromatic performance of the trifocal models. For instance, the LCA of the PanOptix at the far focus (0th order) was slightly lower than that of the Trinova (− 1st order) despite a substantial difference in their Abbe numbers. Note that at the 0th order, IOL's LCA is not affected by diffraction. On the other hand, Trinova's intermediate-point (0th order) LCA was close to that of the AT Lisa's LCA at far (0th order), which can be again explained by similarities in their material properties. The Trinova's higher far-point LCA can be understood, given its design that utilizes a − 1 order, which effectively increases LCA. But conversely, the LCA correction takes place in the + 1 order. This increase or decrease is add-power (P_0_) related and can be quantified using the following formula:

$$P\left( \lambda \right) = mP_{0} \frac{\lambda }{{\lambda_{0} }} \left[ D \right]$$

where λ and λ_0_ are the measured and designed wavelength, respectively, and *m* is the diffractive order. For example, if Trinova's λ_0_ = 550 nm and the add power is 1.80D, and after assuming the applied spectral conditions, then P(λ = 480 nm) = 2.06D and P(λ = 644 nm) = 1.54D, which gives an LCA of 0.52D. Since a diffractive optical element (DOE) has a negative sign of LCA, the material-induced LCA (0.47 ± 0.00D) is nearly fully compensated. At the far focus, however, a negative diffractive order cancels out the negative sign of DOE's dispersion, which results in an LCA increase by the same amount. Taking into account a material contribution of 0.47D, the theoretical far LCA of the Trinova would be 0.99D, which is close to 0.96D measured.

The Triumf utilizes positive non-zero orders at all foci, which enables a more effective LCA correction. Synergy's optical behavior points to a similar diffractive approach. Still, chromatic-aberration levels differ between the two models, i.e., Synergy produces negative LCA at all foci while Triumf's far focus LCA is positive (albeit close to zero) but negative at intermediate and near points. One potential explanation for the observed difference is the higher Abbe number of the Synergy lens. The Triumf's dispersion properties are minimally better than that of the AcrySof material, which yields an excess LCA of about 0.92D. The measured value of the Triumf at the far focus lies at 0.10D, indicating an effective achromatization. However, given the lower dispersion of the Tecnis material, a negative value can be produced at far, which can also compensate for the LCA of the human eye. This correction becomes more effective with higher orders and adds powers, which results in minimal differences in the polychromatic vs. monochromatic optical quality despite a 1D LCA contribution of the model eye.

The 1st/2nd order design has a drawback, i.e., increased spectral dependency of the optical performance^8,12,13^. The Synergy exhibited a substantial reduction of the optical quality at the intermediate-near range in red light, which is particularly apparent after the inspection of the simulated-VA figure. On the other hand, the comparison of the MTF at 50 lp/mm did not demonstrate a substantial difference indicating that in the case of the Synergy, lower spatial frequencies are more affected by this spectral effect. By contrast, Triumf's performance was altered at all spatial frequencies when the deviation from a designed wavelength increased. The noted reduction of the discrete MTF value ranged from 3.6- to 4.4-fold, which resulted in a large difference between the 480- and 644-nm conditions of approx. four lines (logMAR) at the intermediate focus. The simulated VA reduction from the polychromatic VA level exceeds two lines at far focus (blue light) and over one line at the intermediate and near position, which may have functional implications. Interestingly, Triumf's polychromatic performance remained good, as, potentially, a lower contribution of longer wavelengths to the intermediate and near foci is compensated by improved efficiency in shorter wavelengths. The opposite scenario takes place at the far focus. One may wonder if such a difference in the spectral composition of the retinal image could also affect the patients' color perception.

In an earlier study by our group, the clinical impact of the wavelength-dependent optical-quality change was assessed in patients after Tecnis Symfony (Johnson and Johnson Vision) implantation^12^. The Symfony also uses the 1st/2nd order approach to extend the depth of focus in pseudophakia. In that interdisciplinary research, the lens performance was first assessed in the laboratory based on the MTF principle demonstrating the far-dominant performance in longer wavelengths and the expense of the intermediate focus. Although the clinical evaluation did not improve patients' best-corrected distance VA, perhaps because of a limiting neural factor, distance-corrected intermediate and near VA were significantly reduced by 0.06 logMAR and 0.09 logMAR, respectively. In addition, contrast sensitivity was also reduced at three spatial frequencies when the test was performed at 40 cm compared to the standard white-light condition. Those findings revealed that Symfony's spectral dependence impacted VA and contrast sensitivity with a close correspondence between the clinical- and MTF-derived metrics. However, whether the monochromatic performance of the 1st/2nd order trifocal designs translates into a clinical effect, such as decreased visual function or altered color perception, needs to be addressed in a patient-based study.

To compare our laboratory-derived VA with clinical data, we investigated studies assessing the trifocal models examined in our study. The simulated VA outcomes, as presented herein, fall within the range of published clinical results. In a study by Torky et al., visual outcomes were compared after bilateral implantation of three presbyopia-correcting IOLs, including PanOptix, AT Lisa, and Symfony^24^. They reported a binocular defocus curve of 28 patients comprising each IOL group. The results showed that both PanOptix and AT Lisa exhibited a peak at 0 D of defocus, followed by differences in the secondary focus locations; PanOptix demonstrated the second VA peak at − 2.0 D (50 cm), while AT Lisa peaked at − 2.5 D (40 cm)^24^. These PanOptix results align with those recently reported by Dick et al., who conducted a clinical comparison of PanOptix and Synergy IOLs. Notably, Synergy displayed better VA at every defocus point than PanOptix, with intermediate and near peaks recorded at approximately 66 cm and 33 cm, respectively^11^. Similarly, a recent assessment of patients undergoing refractive lens exchanges receiving Synergy IOLs corroborated the findings^25^. Baur et al. reported a preferred intermediate distance of 72.4 ± 6.4 cm and a preferred near distance of 36.9 ± 3.0 cm, which corresponds well with the results of our study^25^. Additionally, Kim et al. conducted a comparative analysis of visual outcomes through a mix-and-match procedure involving the Triumf, FineVision HP, AT Lara, and AT Lisa Tri^26^. Kim et al.'s findings validated the anticipated VA decline with defocus; however, the intermediate focus of the Triumf position appeared to flatten without a distinct secondary peak. Moreover, their assessment reflected the predicted reduction in VA at near compared to intermediate distances, exemplified by a logMAR VA of approximately 0.18 at − 1.50 D, which was reduced to 0.23 at − 2.50 D^26^.

In conclusion, each trifocal IOL utilizes a unique diffractive design, each alternating LCA differently. On the one hand, the selection of the diffractive orders (− 1, 0, and + 1) yielded higher LCA levels at the far and intermediate foci. On the other hand, this design demonstrated a lower (and reversed) spectral-dependency performance in monochromatic light of shorter and longer wavelengths, which contrasted with the 1st/2nd order design. The latter, however, enabled an effective correction of LCA aberration at all three foci. Therefore, trifocal IOLs should be optimized, taking into account these two aspects of spectral performance.

## Data Availability

All data generated or analyzed during this study are included in this published article.
